# Distortions in Development of Intestinal Microbiota Associated with Late Onset Sepsis in Preterm Infants

**DOI:** 10.1371/journal.pone.0052876

**Published:** 2013-01-14

**Authors:** Volker Mai, Roberto Murgas Torrazza, Maria Ukhanova, Xiaoyu Wang, Yijun Sun, Nan Li, Jonathan Shuster, Renu Sharma, Mark Lawrence Hudak, Josef Neu

**Affiliations:** 1 Department of Epidemiology, College of Public Health and Health Professions and College of Medicine and Emerging Pathogens Institute, University of Florida, Gainesville, Florida, United States of America; 2 From the Department of Pediatrics, College of Medicine University of Florida, Gainesville, Florida, United States of America; 3 Department of Microbiology and Immunology, The State University of New York at Buffalo, Buffalo, New York, United States of America; 4 Center of Excellence in Bioinformatics and Life Sciences, The State University of New York at Buffalo, Buffalo, New York, United States of America; 5 Department of Health Outcomes and Policy, College of Medicine, University of Florida, Gainesville, Florida, United States of America; 6 Department of Pediatrics, University of Florida College of Medicine, Jacksonville, Florida, United States of America; Emory University School of Medicine, United States of America

## Abstract

Late onset sepsis (LOS) is a major contributor to neonatal morbidity and mortality, especially in premature infants. Distortions in the establishment of normal gut microbiota, commensal microbes that colonize the digestive tract, might increase the risk of LOS via disruption of the mucosal barrier with resultant translocation of luminal contents. Correlation of distortions of the intestinal microbiota with LOS is a necessary first step to design novel microbiota-based screening approaches that might lead to early interventions to prevent LOS in high risk infants. Using a case/control design nested in a cohort study of preterm infants, we analyzed stool samples that had been prospectively collected from ten preterm infants with LOS and from 18 matched controls. A 16S rRNA based approach was utilized to compare microbiota diversity and identify specific bacterial signatures that differed in their prevalence between cases and controls. Overall α-diversity (Chao1) was lower in cases two weeks before (p<0.05) but not one week before or at the time of diagnosis of LOS. Overall microbiota structure (Unifrac) appeared distinct in cases 2 weeks and 1 week before but not at diagnosis (p<0.05). Although we detected few operational taxonomic units (OTUs) unique or enriched in cases, we found many OTUs common in controls that were lacking in cases (p<0.01). *Bifidobacteria* counts were lower in cases at all time points. Our results support the hypothesis that a distortion in normal microbiota composition, and not an enrichment of potential pathogens, is associated with LOS in preterm infants.

## Introduction

As newborn infants mature, a community of intestinal bacteria begins to establish a commensal and/or symbiotic relationship with the host. In general, this relationship supports several favorable processes that maintain and strengthen the intestinal mucosal barrier. Perturbations in the establishment of bacterial colonization of the intestine in the premature infant with an immature immune response due to lack or delay of enteral feeding, [1]–[4] repeated and often prolonged exposure to broad spectrum antibiotics, and hemodynamic disturbances may initiate a cascade of events resulting in impaired integrity of the mucosal barrier.[1]–[3], [5]–[7] Microbial translocation or increased permeability to microbial components may then initiate a systemic inflammatory response syndrome (SIRS), sepsis, necrotizing enterocolitis (NEC), and multisystem organ failure [5], [8].

High throughput molecular technologies now provide sufficient detail about intestinal microbiota to allow novel insights into the nature and implications of deviations of intestinal bacterial colonization in preterm infants from normal patterns in healthy infants. The latest technologies facilitate the characterization of abnormal patterns of usual commensals as well as the identification of previously undiscovered microbial signature sequences that might constitute novel pathogens. The normal ontogeny of intestinal microbiota in premature infants, including distinguishing normal from aberrant microecology, has not been elucidated. Previous studies using high throughput sequencing methods from our group have shown that meconium in preterm infants contains microbial DNA, [9] that the diversity of microbes in preterm infants increases over time, and that the microbial ecology of the gut in infants who subsequently develop NEC differs from that of closely matched controls [10].

Late-onset sepsis (LOS) is typically defined as identification of pathogenic organisms from blood culture acquired after the third day of life and represents a common complication of prematurity. [11] Coagulase-negative *Staphylococci* (CONS) are the most prevalent microbes followed by gram-negative organisms which have greater mortality (19%–36%) than CONS. [11] The lack of enteral nutrient and the need for prolonged indwelling catheters and other invasive foreign bodies increase the risk for infections caused by Gram positive organism especially *Staphylococci*, but the notion that many of these cases can result from translocation of bacteria through the intestinal mucosal barrier has become a tenable alternative hypothesis. [5] Previous studies using non-culture based techniques in preterm infants have shown a prevalence of *Staphylococcus* in the feces of these infants [12] and provide feasibility to the proposition that a number of the septic bacteremia cases seen in these infants are of intestinal origin.

The purpose of this study was to compare the evolving intestinal microbiota in premature infants born at ≤32 weeks gestation who developed culture proven LOS to matched uninfected control infants using a culture-independent 16S rRNA based approach.

We hypothesized that compared to control infants, infants with sepsis would have significantly different microbial colonization patterns. We further hypothesized that differences in the use of antibiotics prior to onset of LOS, the time of onset of feeding, and the type of feeding (formula vs. maternal milk) could be correlated with observed differences in the developing microecology.

## Materials and Methods

### Ethics Statement

Written informed consent was obtained from the infants’ parents and investigations were conducted according to the principles expressed in the Declaration of Helsinki. The study including consent procedure was approved by the UF/Shands Institutional Review Boards.

### Patients

Premature infants born at ≤32 weeks postmenstrual age were enrolled at three University of Florida affiliated hospitals shortly after birth once parents consented. Infants born before 23 weeks postmenstrual age or with major congenital anomalies or malformations were excluded. LOS was defined as the identification of a blood culture obtained after 3 days of age that was positive for a pathogenic organism in an infant with one or more of the following signs: temperature instability, leukocytosis or neutropenia, increased immature to total (I/T) neutrophil ratio, or elevated C-reactive protein. Two control infants were selected and matched to each LOS case infant by age (+/− one week), birth weight, hospital of birth, and date of birth (+/−2 months). We couldn’t match an appropriate second control infant for two of the cases, resulting in a total of 18 control and 10 case infants.

### Samples

Weekly stool samples from study infants were collected from diapers beginning with the first stool (meconium) and continuing until discharge, for immediate storage at −80°C. The samples analyzed from cases included those collected 2 weeks before LOS (mean ± SD; 15.1±4.6 days prior to LOS), 1 week before LOS (7.4±4.6 days prior), and the sample closest to diagnosis of (2.8±3.1 days prior) LOS. Samples from matched control infants were chosen during the same week of life at which the samples from the cases were obtained.

### Microbiota Analysis

#### DNA extraction and quality control by denaturing gradient gel electrophoresis (DGGE)

DNA was extracted from 200–300 mg fecal samples using a modified Qiagen stool DNA extraction protocol that included a bead beating step. [13] We used DGGE analysis of the V6–V8 region as described previously for initial quality control. [3].

#### 16S rRNA sequence analysis

DNA was amplified using a primer set based on universal primers 27F (AGAGTTTGATCCTGGCTCA) and 533R (TTACCGCGGCTGCTGGCAC) to which titanium adaptor sequences and barcodes were added. Cleansed PCR products were pooled in equimolar amounts and submitted for sequencing using 454-Titanium chemistry. From the resulting raw data set, low quality sequences or sequences with a length less than 150 nucleotides were removed. We used the ESPRIT-tree algorithm, which maintains the binning accuracy of ESPRIT [14] while improving computational efficiency to bin sequences into Operational Taxonomic Units (OTUs) using similarity levels from 99% (species/strain level) to 80% (phylum level). We used QIIME [15] to calculate Chao rarefaction diversity and UniFrac distances [16] for comparing α and β diversity respectively.

#### qPCR analysis

We used a *Bifidobacteria* specific primer set (F: 5′ TCG CGT C(C/T)G GTG TGA AAG 3′; R: 5′ CCA CAT CCA GC(A/G) TCC AC 3′, annealing temperature 58°C) to quantify the amounts of *Bifidobacteria* genome equivalents in fecal samples. Duplicate vials containing 10 ng of DNA were included in each reaction and DNA purified from *B. longum* was used to generate the standard curve. Samples with less than one genome equivalent/ng of DNA were considered as negative.

### Statistics

Each case was matched to evaluate demographic data and clinical characteristics. Conditional case-control odds ratios (OR) were calculated by exact Mantel-Haenszel method for matched proportions, stratifying by case-control matching. The QIIME package was used to calculate *p*-values for differences in UniFrac distances. To test for microbiota differences in the abundance of OTUs a paired chi square test was followed by Fisher combining. This approach was necessary as most of the OTUs were only detected in a few samples, preventing a matched analysis. We adjusted for an expected high false discovery rate by increasing the requirement for statistical significance to *p*<0.01.

## Results

### Clinical Characteristics

The mean postmenstrual age in case and control infants was 27 weeks (range, 25 to 31 weeks). Mean birth weight was 983±333 grams in cases and 1044±338 grams in controls. There were 2 controls each for 8 cases and only 1 control each for 2 cases. There were no significant differences in clinical characteristics between the two groups except for type of exclusive enteral feeding (maternal milk *vs.* formula) and amounts of milk consumed. 90% of the infants in the sepsis group received predominantly maternal milk compared to 39% of controls (p<0.05). However, 72% of the controls at least received some maternal milk. In the sepsis group 40% received human milk fortifier compare to 22% in the control group (Table 1).

**Table 1:** Baseline Characteristics of the Infants. (Mean ± SEM).

Characteristic	Sepsis (N = 10)	Control (N = 18)
**Birth weight – g**	983±105	1044±79.7
**Postmenstrual age at birth – wk**	27±0.43	27±0.34
**Male sex – no. (%)**	7/10 (70)	8/18 (44)
**Mode of delivery – no. (%)**		
Vaginal	4/10 (40)	3/18 (17)
C-Section	6/10 (60)	15/18 (83)
**Type of Milk – no. (%)**		
Maternal milk exclusively/predominantly	9/10 (90)	7/18 (39)*
Formula exclusively/predominantly	1/10 (10)	5/18 (28)
Both	0/10	6/18 (33)
Any maternal milk	9/10 (90)	13/18 (72)
Human milk fortifier	4/10 (40)	4/18 (22)
Volume of milk consumed (ml/kg/d)	98.21±17.37	132.51±9.87*
**Day of Life of first feed**	3.0±0.5	2.5±0.3
**Day of Life of diagnosis**	25±4	
**Maternal use of antenatal corticosteroids no. (%) no./total no. (%)**		
Any	2/10 (20)	5/18 (28)
Full course	4/10 (40)	7/18 (39)
**Prenatal maternal antibiotic exposure**	7/10 (70)	13/18 (72)
**Apgar score at 1 min**	4.8±0.5	4.2±0.6
**Apgar score at 5 min**	7.2±0.4	6.8±0.5
**Positive pressure ventilation (bag and mask)**	10/10 (100)	15/18 (83)
**Continuous positive airway pressure (CPAP)**	1/10 (10)	3/18 (17)
**Intubation and mechanical ventilation**	8/10 (80)	15/18 (83)
**Antibiotic exposure prior to diagnosis – no. (%)**		
Antibiotic exposure, Yes	10/10 (100)	15/18 (83)
less than 48 hrs	9/10 (90)	11/18 (61)
more than 7 days	5/10 (50)	6/18 (33)
Total days on antibiotics (mean ± SEM)	8.9±1.4	6.5±1.1

* p<0.05.

### Major Clinical Outcomes

There were no significant differences in other morbidities between the two groups, except for a higher incidence of patent ductus arteriosus (PDA) in the LOS group; p = 0.02 (Table 2).

**Table 2:** Major Outcomes.– no./total no. (%).

	Sepsis (N = 10)	Control (N = 18)	P value OR (95% CL)
Intraventricular hemorrhage	2/10 (20)	6/18 (33)	0.56 0.7 (0.1–3.2)
Patent ductus arteriosus	7/10 (70)	4/18 (22)	0.011 7.2 (1.1–47)
Retinopathy of prematurity	4/10 (40)	6/18 (33)	0.56 1.7 (0.3–9.8)
Periventricular leukomalacia	1/10 (10)	0/18	0.16 -
Bronchopulmonary dysplasia	5/10 (50)	5/18 (28)	0.11 6.0 (0.6–65)

OR = ”conditional case-control odds ratio” is by exact Mantel-Haenszel method for matched proportions, stratifying by case-control matching.

### Microorganisms Isolated and Antibiotic Use

CONS was the cause of LOS in 70% of cases. One infant grew both *Enterococcus faecalis* and *Enterobacter cloacae* concurrently (Table 3). All of the cases and 83% of the controls received one or multiple antibiotics at some point during the study. Ampicillin and gentamicin were used in more than 90% of the infants in both groups (Table 4). A prolonged course of antibiotics for more than 7 days was observed in 50% of the LOS infants compared to 33% control infants (Table 1).

**Table 3:** Bacteria isolated from infants with sepsis.

Blood Cultures	Sepsis (N = 10)
Coagulase-negative *Staphylococcus* (CONS)	7/10 (70)
*Enterococcus faecalis*	2/10 (20)
Group B *Streptococcus*	1/10 (10)
*Enterobacter cloacae*	1/10 (10)

**Table 4:** Antibiotic exposure. – no./total no. (%).

	*Sepsis*	*Control*
**Ampicillin**	10/10 (100)	16/18 (88)
**Gentamicin**	10/10 (100)	16/18 (88)
**Vancomycin**	10/10 (100)	4/18 (22)
**Oxacillin**	3/10 (30)	1/18 (5)
**Piperacillin-tazobactam**	3/10 (30)	3/18 (16)
**Azithromycin**	1/10 (10)	2/18 (11)
**Cefotaxime**	1/10 (10)	1/18 (6)
**Nafcillin**	3/10 (30)	0/18 (0)

### Microbiota Analysis

After processing and screening of specimens, we analyzed a total of 180,845 sequences that represented an average of 1,773 sequences per sample. The average length per sequence was 477 nucleotides (range 200–535).

**Figure 1:**
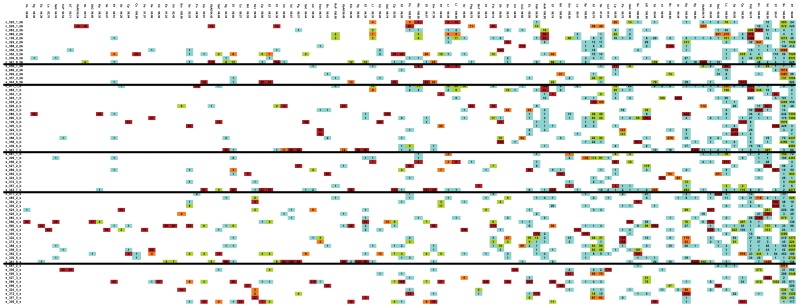
Heatmap showing the distribution of the most prevalent OTUs at 98% similarity level. List of full names for closest matches shown by abbreviation in Figure 1 are listed below: ArActinomyces_radingae AsNAchromobacter_sp._N2 bCbacterium_C20 BdBacteroides_dorei BdBacteroides_dorei bDbacterium_DR304 BsCBacteroides_sp._CB53 BsTBosea_sp._TPR12 bTbacterium_Te14R BuBacteroides_uniformis BvBacteroides_vulgatus CdClostridium_disporicum CfCitrobacter_freundii CkCitrobacter_koseri ClClostridium_lavalense CpClostridium_perfringens Cs1Clostridium_sporogenes Cs2Clostridium_sordellii Cs3Clostridium_sp._75064 CsCClostridium_sp._CS4 CsPClostridium_sp._PN6–15 EaEnterococcus_avium EcEnterobacter_cancerogenus EcEscherichia_coli Ef1Enterococcus_faecalis Ef2Enterococcus_faecium EmEnterococcus_malodoratus EsmEnterobacter_sp._mcp11b FmFinegoldia_magna FnFusobacterium_nucleatum KoKlebsiella_oxytoca KpKlebsiella_pneumoniae LaLactobacillus_animalis LcLeuconostoc_citreum LlLactococcus_lactis LpLactococcus_plantarum LrLactococcus_raffinolactis Pa1Propionibacterium_acnes Pa2Pantoea_agglomerans PgPropionibacterium_granulosum PsEPhyllobacterium_sp._EBBLQ01 PsgPeptoniphilus_sp._gpac121 PsHPropionibacterium_sp._H456 RsPRhodococcus_sp._PN8 Sa1Staphylococcus_aureus Sa2Streptococcus_agalactiae SeStaphylococcus_epidermidis Sg1Serratia_grimesii Sg2Streptococcus_gallolyticus ShStaphylococcus_hominis SmSerratia_marcescens SmStenotrophomonas_maltophilia SpStaphylococcus_pasteuri SsStreptococcus_salivarius Ss1Streptococcus_sp._10aVMg3 SsCStaphylococcus_sp._C-12 SsCSerratia_sp._CJB2 SsGStreptococcus_sp._G3 SvStreptococcus_vestibularis VaVeillonella_atypica VbAVeillonellaceae_bacterium_ADV_07/08/06-B-1388 VdVeillonella_dispar WcWeissella_confusa.

We then clustered sequences using ESPRIT-tree at 98%, 95% and 90% similarity levels to obtain OTUs containing similar sequences for further microbiota analysis. We have previously shown that, compared to other commonly used algorithms, ESPRIT-tree improves binning accuracy while reducing computational requirements [17]. We removed OTUs that contained less than five sequences to reduce loss of statistical power due to multiple comparisons, retaining 2636, 365 and 184 OTUs at the respective similarity levels for analysis. The distribution of the most dominant OTUs at the 98% similarity level is shown in Figure 1. Most of the dominant OTUs are detected at similar frequencies in cases and controls. While some dominant OTUs, such as PsE (with closest match to the α-proteobacterium *Phyllobacterium*) are almost absent from the cases, none of the dominant OTUs are significantly more prevalent in cases.

**Figure 2:**
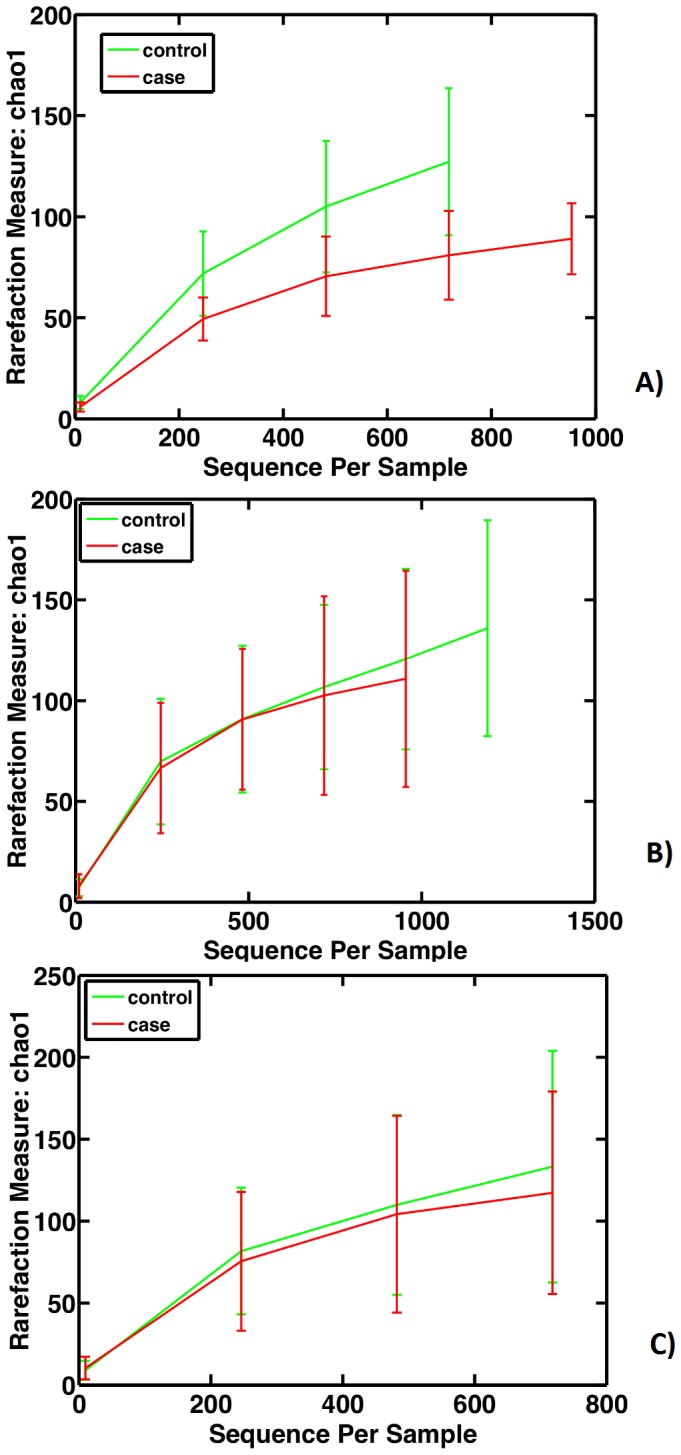
Chao rarefaction diversity. Chao diversity was calculated from sequence distribution A) 2 weeks before; B) 1 week before; and C) within 72 hours of LOS diagnosis. Error bars indicate SE.

**Figure 3:**
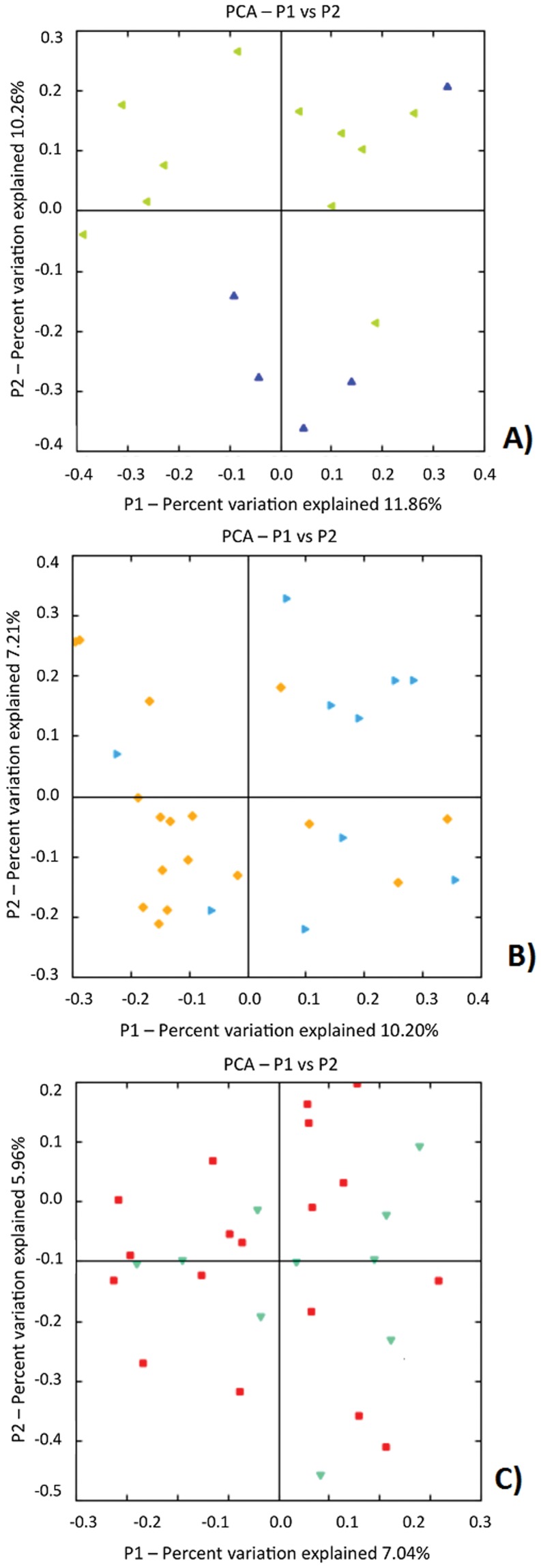
Unifrac diversity measures. Principal component analysis (PCA) of overall diversity based on UniFrac (unweighted) metric A) 2 weeks before; B) 1 week before; and C) within 72 hours of LOS diagnosis Squares represent controls and triangles represent cases. P1 is component 1 and P2 component 2.

Using these binned sequence data, we first analyzed measures of within (α) and between (β) sample diversity. The predicted total numbers of OTUs present, estimated using Chao1-based rarefaction curves (a common measure of diversity in populations that reflects α-diversity) did not differ between cases and controls at any of the three time points (Figure 2A–C). When we analyzed overall microbiota structure using the UniFrac metric (unweighted), which is based on branch length differences between the case/control groups in a phylogenetic tree generated from all 16S rRNA sequences, we detected that 2 weeks before LOS-diagnosis four of the five available samples from cases formed a cluster distinct from controls (Figure 3A). This clustering was weaker in the samples collected one week before LOS-diagnosis (Figure 3B) and was not detected closer to diagnosis (Figure 3C).

**Figure 4:**
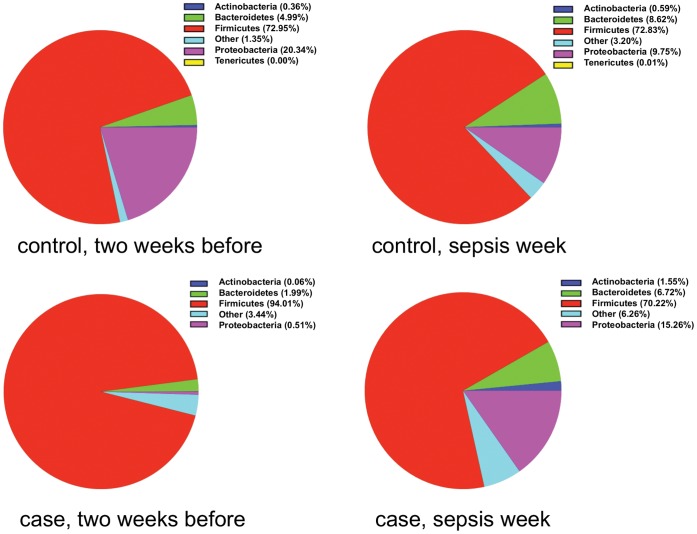
Changes in proportion of bacterial phyla. Proportions of the four major phyla 2 weeks before and within 72 hours of LOS diagnosis.

We next analyzed differences in the proportion of bacterial groups at the phylum level. Four phyla (*Firmicutes, Proteobacteria, Bacteroidetes* and *Actinobacteria*) dominated the microbiota in most samples (Figure 4). Two weeks before diagnosis the proportion of *Proteobacteria* was much lower in LOS (0.5%) than in control infants (20%). While the proportion of *Proteobacteria* in controls went down to 9.75%, cases experienced a bloom in *Proteobacteria* to 15% around the time of diagnosis.

**Figure 5:**
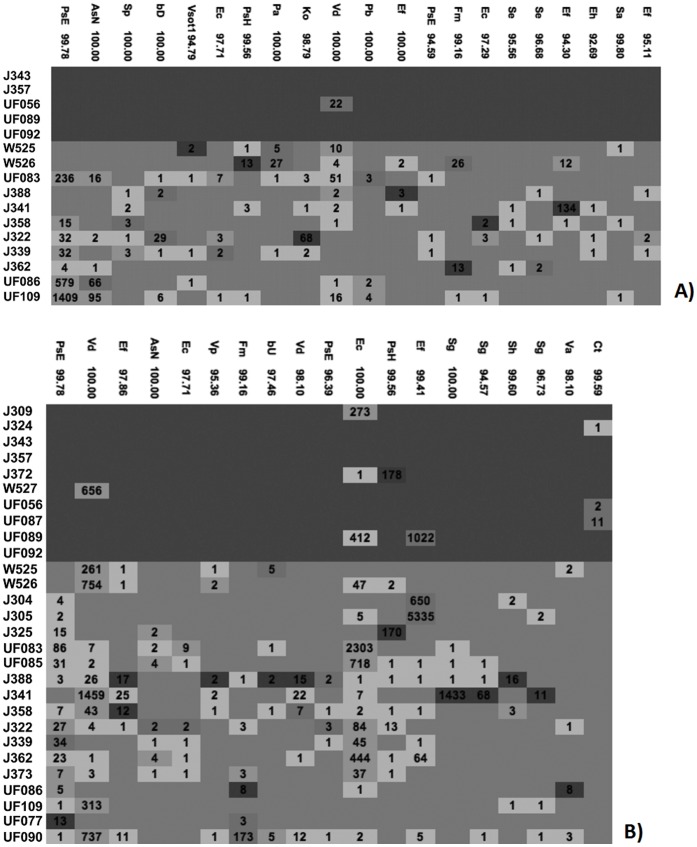
Differences in OTU abundance. Heat map of selected OTUs at 98% similarity by subject A) 2 weeks before and B) within 72 hours of LOS diagnosis. Sepsis cases are shown on the top (dark blue shaded area) and controls in the bottom (light blue shaded area). Cell Colors indicate the ratio for a particular OTU in a sample to the average ratio of this OTU in all samples. Dark red: >10, light red: >5 and < = 10, light green: >1.5 and < = 5 and light blue: < = 1.5. Numbers indicate how often the OTU was detected. List of full names for closest matches shown by abbreviation in Figure 5 are listed below: AsN Achromobacter_sp._N2 Bb Bifidobacterium_breve Cp Clostridium_perfringens Cs Cronobacter_sakazakii CsC Clostridium_sp._CS4 Ct Corynebacterium_tuberculostearicum Ea Enterococcus_avium Ec Escherichia_coli Ec Enterobacter_cloacae Ef Enterococcus_faecalis Ef Enterococcus_faecium Ef Escherichia_fergusonii EsC Enterobacter_sp._CTSP23 Fm Finegoldia_magna Ko Klebsiella_oxytoca Kp Klebsiella_pneumoniae Lc Leuconostoc_citreum Pb Pseudomonas_brenneri Pm Phyllobacterium_myrsinacearum PsE Phyllobacterium_sp._EBBLQ01 Sa Streptococcus_agalactiae Se Staphylococcus_epidermidis Sg Streptococcus_gallolyticus Sh Staphylococcus_hominis Sm Stenotrophomonas_maltophilia Sp Staphylococcus_pasteuri Ss1 Streptococcus_sp._10aMclG2 Sv Streptococcus_vestibularis Va Veillonella_atypica VbA0 Veillonellaceae_bacterium_ADV_07/08/06-B-1388 VbA1 Veillonellaceae_bacterium_ADV_12/01/04-B-1195 Vd Veillonella_dispar Vp Veillonella_parvula.

We failed to detect differences between the two groups in the prevalence of OTUs representing bacteria present in the blood of LOS cases. Two weeks before diagnosis we did not detect any OTUs with statistically significantly increased prevalence in cases (Figure 5A). However, around the time of diagnosis we detected one OTU, matching closely (99.5%) to *Corynebacterium tuberculostearicum*, that was more prevalent in cases than in controls (Figure 5B). In contrast, we detected multiple OTUs frequently in controls that were never or only rarely detected among cases.

**Figure 6:**
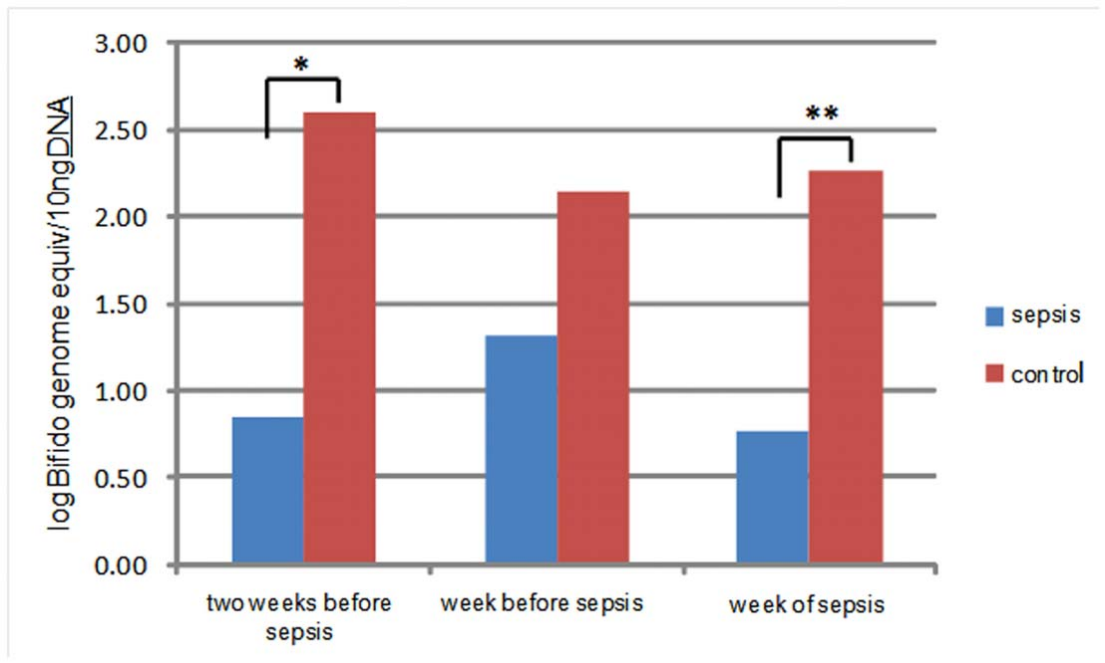
qPCR for fecal counts of Bifidobacteria. * p-value 2 weeks before LOS - 0.05; **p-value week of sepsis –0.03.

To quantify differences in the levels of Bifidobacteria in our samples we used qPCR (Figure 6). This was necessary as the ‘universal’ primer set used for the sequence analysis is biased against *Bifidobacteria*. We observed lower numbers of Bifidobacteria in cases compared to controls. This observation was significant at two of the time points when levels of *Bifidobacteria* were more than 10-fold higher in controls.

## Discussion

The role of the intestinal commensal microbiota in the maintenance of human health is receiving increased research interest. [18] Although feces are not an ideal sample of intestinal microbiota, they can be collected non-invasively and contain up to 1×10^12^ bacteria/g [19] in a composition thought to be similar to that present in the proximal colon. Although distortions in microbiota composition and function have been correlated with a variety of disease states causal associations have rarely been investigated. [20] This is mainly due to the difficulty in prospectively collecting and analyzing microbiota samples in the disease free individual, because microbiota diversity is likely to be strongly affected by various disease states and treatment modalities. Our study was able to benefit from a prospective cohort design, which allowed us to analyze fecal samples collected before the onset of clinical disease.

While the intestinal microbiota development at a molecular level has already been well documented in animal models to correlate with gastrointestinal (GI) tract development, mucosal integrity, and even nutritional status, these correlations have only recently begun to emerge for human term infants. [21] Even less is known about the longitudinal development of microbiota in preterm infants. Such infants are born with an immature gastrointestinal tract [22], and they are often cared for in the neonatal intensive care unit that is not a conducive environment for normal microbiota colonization. Various groups, including ours, have previously reported microbiota distortions associated with NEC. [4], [22].

Our matched case control analysis identified microbiota differences in very premature infants with LOS. Consistent with many reports, CONS was the most common microbe cultured from the blood of infants with LOS. Although we were not able to detect a difference in the abundance of CONS in the stool samples of LOS and control infants; we detected a higher proportion of organisms in the *Firmicutes* phylum (the phylum to which CONS belongs) in LOS cases 2 weeks before diagnosis (Figure 3).

Similar to our earlier report which suggested similar number and evenness of OTUs (α-diversity) in NEC and control infants, we did not detect differences in α-diversity in feces collected before and near the time of LOS diagnosis between LOS cases and controls (Figure 2). In contrast, microbiota structure, which measures the similarity of OTUs detected in both groups (using a Unifrac-based PCO analysis), was different in LOS cases for the two time points before but not at the time point closest to diagnosis (Figure 3).

Our findings suggest that the types and distributions of bacteria that initially colonize the intestine in premature infants differ in LOS compared to uninfected control babies. Our observation that *Proteobacteria* were low at early time points in LOS cases compared to controls supports that finding. The bloom in *Proteobacteria* that we detected in cases at the time of diagnosis is similar to our prior report of such a bloom in NEC cases. (10) Research by others has shown similar blooms in *Proteobacteria* prior to the onset of exacerbations of inflammatory bowel disease. [23] Infants have a bias towards Th2 responses and an immature but often exaggerated Th1 response. Immature intestinal response and alterations or abnormal pattern of colonization of the intestinal microbiota, predispose the intestine of neonates to inflammation and to a cascade of pro-inflammatory and counter-inflammatory cytokines response. Based on this consistent finding, we propose that a delay in colonization by *Proteobacteria*, which is normal and immunologically well-tolerated during the initial weeks of microbiota development, might result in an excessive immune response that compromises the integrity of the mucosal barrier, thereby allowing translocation of bacteria into the circulation resulting in LOS, NEC, and extensive inflammation. The specific mechanisms linking the intestinal microbial changes to sepsis remain unclear, but recent studies implicating disruption of the normal intestinal microbiota and induction of inflammasome are intriguing as potential mechanisms. [24], [25].

Our observation that many OTUs frequently detected in controls were not detected in LOS cases suggests that a lack of colonization by various normal or non-pathogenic bacteria, rather than the presence of a pathogen, might increase the risk of LOS. Many factors including diet, mode of delivery and antibiotics can influence intestinal bacterial colonization. The difference in feeding that we observed between cases and controls, with a higher proportion of maternal milk intake but lower total volume intake among the cases, likely contributed to the differences in microbiota. In our study there was no statistical difference in mode of birth between cases and controls. 90% of infants in the sepsis group received antibiotics in the first 48 hrs of life compared to 61% in the control group. By the time of sepsis almost all infants received antibiotics.

We detected only a single OTU that was statistically significantly more prevalent in cases than in controls. This OTU, which matched closest to *Corynebacterium tuberculostearicum*, was detected in three out of ten cases only near the time of diagnosis of LOS and in none of the controls at any time point. *Corynobacteria*, including *tubercolostearicum,* have been isolated from patients with mastitis. [26] We were not able to ascertain whether the mothers of these infants had mastitis. At this time we can only speculate that colonization with this organism may have contributed to the pathogenesis of disease in some LOS cases, since this microbe was not detected in the blood cultures of LOS cases.

In addition to the 16S sequencing, we also evaluated *Bifidobacteria* levels using qPCR. We performed this analysis due to the known bias against this genus of the primer set we used for sequencing. The lower concentrations of this genus in babies who subsequently developed sepsis is interesting in light of some of the known “probiotic” properties ascribed to this genus. Using culture based techniques, previous studies have demonstrated that *Bifidobacteria* colonize the healthy newborn intestine soon after birth and likely contribute to normal intestinal development. [27].

PDA has been linked to an increased need for mechanical ventilation, to chronic lung disease, to intraventricular hemorrhage, and to necrotizing enterocolitis. [28], [29] In this study we detected a significantly higher rate of PDA in the sepsis group. This is not surprising since i) infants that develop sepsis often require higher use of colloids and fluid resuscitation, which can initiate or exacerbate PDA and ii) babies that develop sepsis frequently also develop some pulmonary hypertension and PDA.

Our prospective cohort study is limited to 3 hospitals in one specific geographical region and so cannot be generalized to a larger population. Other studies, such as those currently ongoing at other US locations and around the world, will need to confirm our findings. While analyses of associations between individual OTUs and disease risk are prone to detect false positives, due to the multiple analysis issue, our findings of the delayed *Proteobacteria* colonization and the lack of multiple OTUs from the microbiota in LOS cases are robust. The hypothesis that delayed colonization by *Proteobacteria* increases disease risk in preterm infants (for both NEC and LOS) needs to be further tested, as its confirmation would have important implications for designing effective early detection and prevention approaches.
